# Knowledge, attitude, and practice towards mental illness service provision and associated factors among health extension professionals in Addis Ababa, Ethiopia

**DOI:** 10.1186/s13033-019-0261-3

**Published:** 2019-01-28

**Authors:** Eftu Ahmed, Hailu Merga, Fessahaye Alemseged

**Affiliations:** 1 UNICEF, Nairobi, Kenya; 2 Department of Epidemiology, Institute of Health, Jimma University, Jimma, Ethiopia

**Keywords:** Mental illness service provision, Knowledge, Attitude, Practice, UHEPs

## Abstract

**Background:**

Mental, neurological and substance use disorders are highly prevalent in Ethiopia which are known to result in substantial disability. Improving the knowledge, attitude and practice of the primary health care workers is important to reduce this problem. Hence, this study aimed at assessing knowledge, attitude, and practice towards mental illness service provision and associated factors among urban health extension professionals (UHEPs) of Addis Ababa City Administration.

**Methods:**

A cross sectional study design was used. Data was collected from 455 study participants using structured and pre-tested self-administered questionnaire and analyzed using SPSS version 20 software respectively. Multivariate logistic regression analysis was performed to identify variables which have significant association with the outcome variables. The level of significant association was determined by adjusted odds ratio (AOR) with 95% confidence interval.

**Results:**

This study showed that 44.0% of urban health extension professionals (UHEPs) had adequate knowledge, 93.4% did not have positive attitude and 75.2% had good practice towards mental illness. Age 30 years and above [adjusted odds ratio (AOR): 95% CI 0.55 (0.34, 0.90)], having diploma educational status [AOR 95% CI 0.49 (0.32, 0.78)], and personal history of mental illness [AOR 95% CI 0.10 (0.01, 0.89)] were found to have a negative association with knowledge. Presence of job aid (AOR 95% CI 4.30 (2.59, 7.15)) and having good knowledge (AOR 95% CI 0.52 (0.32, 0.85) were increased the practice of service provision of UHEPs.

**Conclusion:**

Less than half of UHEPs had adequate knowledge, most had unfavorable attitude and about three-fourth of them had good practice. Presence of job aid and having good knowledge were increased the UHEPs practice of mental health service provision. Hence, providing refresher training to UHEPs and fully implementing the national mental health strategy as well as proper clinical supervision and support to improve behavioral change is vital.

## Background

Mental Health, as defined by the World Health Organization (WHO), is a state of well-being in which every individual realizes his or her own potential, can cope with the normal stresses of life, can work productively and fruitfully, and is able to make a contribution to her or his community [1]. Mental, neurological, and substance use (MNS) disorders are prevalent in all regions of the world and are major contributors to morbidity and premature mortality. Evidence showed that 14% of the global burden of disease, measured in disability-adjusted life years (DALYs), can be attributed to MNS disorders [2].

According to WHO Mental Health Atlas 2014, more than 45% of the world population live in a country where there is less than one psychiatrist for every 100,000 people. However, relying solely on specialists to provide services for people affected by mental, neurological and substance use or disorders would prevent millions of people from accessing the services they need [3]. Hence, WHO has proposed the development of community mental health services through integration of mental health into the existing primary health care system and mobilization of community resources [4].

In Ethiopia, mental illness is the leading non-communicable disorder in terms of burden. According to WHO Country Cooperation Strategy (CCS) 2012–2015, in Ethiopia there is an increasing trend of mental health problems. This is due to urbanization affects mental health through the influence of increased stressors and factors such as overcrowded and polluted environment, high levels of violence, and reduced social support. In order to overcome this, WHO and the Ethiopian Federal Ministry of Health (FMOH) are currently implementing the WHO Mental Health Hap Action Program (mhGAP) aimed at integrating mental health into PHC through a decentralization process. Mental Health Gap Action Program (mhGAP) is WHO program aims at scaling up services for mental, neurological and substance use disorders for countries especially with low and middle income [5].

In 2003, FMOH launched a new innovative health care plan, the Health Extension Program (HEP) for accelerated expansion of primary health care coverage. The HEP professionals are female diploma graduate nurses trained on the Health Extension Program for 3 months. While many urban interventions are similar to the rural program, some key differences from the rural setting include the prevention and control of non-communicable disease, mental health, and violence and injury prevention, as these are expected to affect urban populations more significantly. Urban health extension professionals are providing services on mental health promotion and mental illness prevention activities, awareness-raising and anti-stigma campaigns, early detection of mental illness, referral and the ongoing needs of persons with severe mental illness [6].

The urban health extension professional provides service to 500 households and, schools and youth centers in the area. In order to address their knowledge and attitude gap to integrate the mental health into primary health care, in 2015 Ethiopian Federal Ministry of Health (FMOH) has developed “The 2nd generation mental health training package for health extension workers” training manual. To the best knowledge of the researcher, there is a serious shortage of information that helps to explain the knowledge, attitude and practice of mental health service provision in country and also in the study setting. Hence, this study aimed to assess the knowledge, attitude, and practice towards mental health service provision and associated factors among urban health extension professionals of Addis Ababa City Administration.

## Methods and materials

An institution based cross sectional study was conducted in Addis Ababa City in five selected sub cities (Kolfe Keranio, Arada, Addis Ketema, Yeka, and Bole) from October 2017 to December 2017. The city has 3,384,569 people living in it according to the 2007 census with an annual growth rate of 3.8%. Currently, according to the annual report of 2015 from the Addis Ababa Regional Health Bureau, there are 6 hospitals and 98 health centers under the City Administration. Out of 98 health institutions, 60 health centers and 9 hospitals (including hospitals under the FMOH) are providing mental health service to the population. This service is provided by health providers who are trained as per the WHO supported mhGAP program. Accordingly, every year these health facilities are providing services to over 6000 peoples [7]. Unlike the rural health extension workers, urban health extension professionals are placed within the woreda (the third-level administrative divisions of Ethiopia) health office.

### Population

The source population was all urban health extension professionals who were working in Addis Ababa City Administration. Whereas, the study population was sampled urban health extension professionals who were working in the selected 44 woredas during the time of data collection and were fulfilled the inclusion criteria. The Urban Health Extension Professionals on long vacation or maternity leave were excluded from the study.

### Sample size determination and sampling procedure

The sample size was calculated by double population proportion formula using Epi-info7.2. The following parameters were considered for the calculation: proportion of Nurses who took training and had good knowledge, 59% and proportion of Nurses who took training and did not have knowledge 40.5%, 84% power, AOR 2.1 and 95% confidence interval [8]. Then, after using design effect of [2] and non- response rate 10%, the final sample size was 506. Multi Stage sampling technique was implemented to select the desired sample. At the first stage, five sub cities were selected out of ten sub cities using simple random sampling. At the 2nd stage, 44 woredas were selected out of the 62 woredas in the selected sub cities using lottery method. Woredas selected were also based on availability of sample to satisfy the desired sample size. At the 3rd stage, out of the total 658 UHEPs, 506 UHEPs were selected from the sampled woredas.

### Measurements

#### Knowledge

In this study knowledge refers to the understanding of mental illness, which includes causes, its symptom (manifestation), treatment, management and risk factor. It was assessed by five questions (four of them were multiple response and one was ‘yes’, ‘no’ and ‘I don’t know’ choices). Respondents who got all correct response have got a maximum of 20 points, higher points indicate good knowledge. Based on total score and distribution, knowledge on mental illness service provision was categorized into inadequate (< 12 scores) and adequate (≥ 12).

#### Attitude

In this study attitude encompasses opinion or perceptions, threat, susceptibility and seriousness that the study participants may have towards mental illness and providing service to person with mental illness. It was assessed by 12 questions. Based on total score and distribution, attitude on mental illness service provision was categorized into unfavorable (< 9 scores) and favorable (≥ 9).

#### Practice

In this study it refers to actions based on knowledge and attitude of the participants in providing mental illness service (diagnosis, referral and counseling) to the community. It was assessed by seven questions. Based on total score and distribution, practice on mental illness service provision was categorized into poor (< 4 scores) and good (≥ 4).

#### Job aid

In this study refers to officially distributed materials (broacher, booklet, flipchart) that can support the UHEPs to make a proper diagnosis, provide better counseling at the point of delivery of service. It was assessed by if they have job aids or not.

### Data collection tools and procedures

Structured and pretested self-administered questionnaire was used as a tool for data collection. The data collection tool was developed and adapted after a thorough review of relevant literature [8–12]. The questioners were grouped into five sections: socio-demographic characteristics, respondent’s knowledge regarding mental illness, respondent’s attitude towards mental illness, respondent’s practice regarding mental illness service provision to the community and factors associated with mental illness service provision.

To ensure the quality of data the questionnaire was first developed in English and translated into Amharic, local language. Then, different individuals fluent in both languages translated the Amharic version back to English to check its consistency. Finally, the instrument was administered in Amharic language. Data collectors and supervisors were trained for a day on how to introduce study tools to the study participants and check the completeness of each questionnaire. Pretest was also conducted on UHEPs similar study participants from kirkos sub cities in Addis Ababa which was not included in the study to check the validity and reliability. Based on the feedback from pretest, possible amendments were made on some parts like changing the choices from one to multiple response possibility. During data collection the supervisor checked the filled questionnaires on daily bases for further checkup. Then, data were checked for the completeness before entry to computer software for analysis.

### Data processing and analysis

The collected data was entered using the Epi-data version 3.1 and analyzed using SPSS version 20.0 statistical software package. The analyzed data was presented in the form of table, fiḡ̄ure and text using frequency and summary statistics such as mean, standard deviation and percentage. Crude and adjusted odds ratio (OR) were calculated to check statistical association between the dependent and independent variables using the bivariate and multivariable analysis. All variables of the study were initially tested for association with knowledge, attitude and practice regarding mental health service provision by using the binary logistic regression model. Those which showed statistical association (*P* < 0.25) in bivariate analysis were putted in the multivariable analysis model to check if the association existed after controlling against all the rest of the variables. A cut off value of 0.25 is supported by literature [13, 14]. All statistical tests were done by assuming 95% confidence interval and statistical significance was considered at P-value less than 0.05.

## Results

### Socio-demographic characteristics of respondents

Out of 506 sample size calculated, a total of 455 participants participated in the study giving a response rate of 89.9%. The mean age of the study participants was 27.9 years, ranged between 20 and 42 years (SD ± 3.94). Among the 455 study participants, 206 (45.3%) were never married and the highest proportion of study participants, 324 (71.3%) were Christian orthodox.

About three-fourth, 74.5%, of the study participants had diploma in terms of their educational status and 85.9% of them were graduated from private institutions. Their mean working experience was 3.71 years (SD ± 2.61) which ranges from 1 month to 10 years (Table 1).

**Table 1:** Socio-demographic characteristics of UHEPs in Addis Ababa, 2017

Variables	Frequency (n = 455)	Percentage (%)
Age group (years)		
20–29	388	74.3
30–44	117	25.7
Mean ± SD	27.94 ± 3.94	
Marital status		
Ever married	249	54.7
Never married	206	45.3
Religion		
Orthodox	324	71.3
Protestant	90	19.8
Catholic	8	1.8
Muslim	33	7.3
Educational status		
Diploma	339	74.5
Degree	116	25.5
Work experience (years)		
≤ 2.5	205	45.1
2.6–5	108	23.7
5–7.5	98	21.5
7.5 and above	44	9.7
Mean ± SD	3.71 ± 2.61	
Institution of graduation		
Private	391	85.9
Government	64	14.1

### Individual factors of urban health extension professionals (UHEPs)

Among the study participants, only 21 (4.6%) had a family history of mental illness while 377 (82.9%), and 57 (12.5%) hadn`t and did not know if there was a history of mental illness within their families respectively. From the study participants, 8 (1.8%) had a personal history of mental illness and 340 (74.7%), and 107 (23.5%) did not have and did not know if they had a personal history of mental illness respectively.

### Knowledge of UHEPs on mental illness

Among the study participants 200 (44.0%) had adequate knowledge regarding mental illness. The four most frequently selected causes of mental illness by the study participants were stress 435 (38.1%), drug/substance abuse 337 (29.5%), weak nerves 142 (12.4%), and brain chemistry 141 (12.3%). Being lonely 356 (17.6%), extreme mood changes 340 (16.8%), and suicidal 316 (15.7%) were the response of the study participants for the manifestation of mental illness. Two hundred thirty-three (50.8%) participants responded provision of drugs, counseling and referral are the way to manage mental illness (Table 2). Respondents were asked for the curability of mental illness and majority of study participants 360 (79.1%) responded mental illness as curable and the rest responded as no and I don`t know.

**Table 2:** Mental illness knowledge among UHEPS in Addis Ababa, 2017

Knowledge assessing question	Frequency (n = 455)	Percentage (100%)
Over all knowledge		
Adequate	200	44.0
Inadequate	255	56.0
Causes of mental illness (n = 1142)^a^		
Stress	435	38.1
Brain chemistry	141	12.3
Week nerve	142	12.4
Drug/substance abuse	337	29.5
Demon/sprit	77	6.7
Don’t know	10	0.9
Manifestation of mental illness (n = 2018)^a^		
Being lonely	356	17.6
Feeling sad	244	12.1
Reduced ability of concentration	240	11.9
Excessive fear or worries	318	15.8
Extreme mood change	340	16.8
Delusion	204	10.1
Suicidal	316	15.7
What is the risk factor of mental illness (n = 1081)		
Stress	418	38.7
Drug and substance abuse	393	36.4
Family history	168	15.5
Sprit possession	38	3.5
Evil sprit	64	5.9
Is mental illness curable (n = 455)		
Yes	360	79.1
No	49	10.8
I don’t know	46	10.1
Management of mental illness (n = 455)^a^		
Provision of drugs	109	24.0
Provision of counseling	81	17.8
Referral	32	7.0
(Provision of drug, counseling and referral)	233	51.2

^a^Multiple responses

### Attitude of UHEPs towards mental illness

More than three-fourth of the UHEPs, 78.5%, did not have a favorable attitude towards mental illness. Among study participants 86 (18.9%) strongly agreed and 171 (37.6%) agreed that people with mental illness are dangerous. Similarly, thirty-nine (8.6%) participants strongly agreed and 183 (40.2%) agreed with mentally ill people are unpredictable, and 62 (13.6%) were not sure. Seventy-six (16.7%) of them strongly agreed and 187 (41.1%) agreed that mentally ill people can lead a normal life. There were 195 (42.9%) who agreed, and 231 (50.8%) who strongly agreed that mentally ill people should be confined to facilities for the rest of their life.

Majority, 284 (62.4%) of the study participants disagreed with the idea of mental illness is a personal weakness; while, only 20 (4.4%) and 64 (14.1%) strongly agreed and agreed respectively. Also, 261 (57.4%) disagreed with mental illness counseling should be left for specialists. One-fifth, 20%, of the study participants strongly agreed and 174 (38.2%) agreed on medication is effective to treat mental illness (Tables 3 and 4).

**Table 3:** Favorable attitude towards mental illness among UHEPs in Addis Ababa, 2017

Characteristics	Frequency (n = 455)	Percentage (100%)
Over all attitude		
Favorable	98	21.5
Unfavorable	357	78.5
Medication is effective for treating mentally ill patients		
Strongly agree	91	20.0
Agree	174	38.2
Undecided	36	7.9
Disagree	140	30.8
Strongly disagree	14	3.1
Mentally patient should be able to receive treatment in all health facilities		
Strongly agree	116	25.5
Agree	143	31.4
Undecided	18	4.0
Disagree	157	34.5
Strongly disagree	21	4.6
People with mental illness can lead a normal life		
Strongly agree	76	16.7
Agree	187	41.1
Undecided	34	7.5
Disagree	130	28.6
Strongly disagree	28	6.2

**Table 4:** Unfavorable attitude towards mental illness among UHEPs in Addis Ababa, 2017

Characteristics	Frequency (n = 455)	Percentage (100%)
People with mental illness are dangerous		
Strongly agree	86	18.9
Agree	171	37.6
Undecided	44	9.7
Disagree	141	31.0
Strongly disagree	13	2.9
Mental illness is sign of personal weakness		
Strongly agree	20	4.4
Agree	64	14.1
Undecided	32	7.0
Disagree	284	62.4
Strongly disagree	55	12.1
Counseling’s of mental illness should be left for specialist		
Strongly agree	46	10.1
Agree	81	17.8
Undecided	35	7.7
Disagree	261	57.4
Strongly disagree	32	7.0
Counseling is unsuccessful for patient with mental illness		
Strongly agree	33	7.3
Agree	35	7.7
Undecided	8	1.8
Disagree	278	61.1
Strongly disagree	101	22.2
Mentally ill patients are usually violent		
Strongly agree	64	14.1
Agree	207	45.5
Undecided	45	9.9
Disagree	120	26.4
Strongly disagree	19	4.2
Mentally ill patients are usually unpredictable		
Strongly agree	39	8.9
Agree	183	40.2
Undecided	62	13.6
Disagree	151	33.2
Strongly disagree	20	4.4
Mentally ill patients need constant care		
Strongly agree	285	62.6
Agree	142	31.2
Undecided	3	0.7
Disagree	10	2.2
Strongly disagree	15	3.3
Mentally ill patients should be confined to facility to the rest of their life		
Strongly agree	8	1.8
Agree	8	1.8
Undecided	13	2.9
Disagree	195	42.9
Strongly disagree	231	50.8
If a person become mentally ill once they easily become ill again		
Strongly agree	22	4.8
Agree	139	30.5
Undecided	49	10.8
Disagree	187	41.1
Strongly disagree	58	12.7

### Reinforcing and enabling factors for mental illness service provision

Among 455 study participants, only 62 (13.6%) were trained during the 3 months training of their being urban health extension professional; while the majority, 311 (68.4%) didn’t know whether they have been trained on mental illness during this time. Almost all, 433 (95.2%) of the study participants were not provided in-service training on mental illness since they have started working as urban health extension.

Almost half of the study participants, 226 (49.7%) had job aids that support the provision of mental illness services. About three-fourth (74.7%) of the respondents claimed that they got supportive supervision, whereas 281 (61.8%) received one to two supportive supervision visits per month (Table 5).

**Table 5:** Reinforcing and enabling factors for mental illness service provision among UHEPs in Addis Ababa, 2017

Characteristics	Frequency (n = 455)	Percentage (100%)
Training on mental health during the 3 months training		
Yes	62	13.6
No	82	18.0
I don’t know	311	68.4
Training on mental health as in-service training		
Yes	22	4.8
No	433	95.2
Having job aids to support mental illness service provision		
Yes	226	49.7
No	229	50.3
Supportive supervision on mental illness		
Yes	340	74.7
No	115	25.3
Frequency of supportive supervision (n = 340)		
One to two times per month	281	61.8
Three to four times a month	59	13.0

### Practice of mental illness service provision

About 98% of the study participants were providing awareness creation of mental illness to families, individuals, and groups, while 10.8% of them were diagnosing mental illness. Most of the study participants 413 (90.8%) were referring (using referral form) clients to further treatment to health centers, general hospitals and hospital that are providing only mental health service. Three hundred ninety-two (86.2%) of the study participants were following up on patients they have referred, whereas 388 (85.3%) of the study participants were providing counseling to mentally ill patients (Table 6).

**Table 6:** Practice of UHEPs towards mental illness service provision in Addis Ababa, 2017

Characteristics	Frequency (n = 455)	Percentage (100%)
Overall practice		
Good	342	75.2
Poor	113	24.8
Provision of awareness creation		
Yes	445	97.8
No	10	2.2
Ever diagnose mental illness		
Yes	49	10.8
No	406	89.2
Refer patient with mental illness		
Yes	413	90.8
No	42	9.2
Provision of counseling to mentally patient people		
Yes	388	85.3
No	67	14.7

### Factors associated with knowledge on mental illness service provision

After bivariate logistic regression analysis; age, religion, educational status, work experience, personal history of mental illness and job aid were candidate variables for multivariate logistic regression analysis with P-value < 0.25.

Among the socio demographic variables, UHEPs age from 30 to 44 years were 45% less likely to have knowledge when compared to those whose age were 20–29 years [AOR = 95% CI 0.55 (0.34, 0.90)]. The other socio demographic variables associated with knowledge was their educational status; those Bachelor degree holders were 51% less likely to have adequate knowledge compared to those UHEPs who had diploma [AOR = 95% CI 0.49 (0.32, 0.78)]. UHEPs who did not have personal history of mental illness were 90% less likely to have adequate knowledge compared to those UHEPs who had personal history [AOR = 95% CI 0.10 (0.01, 0.89)] (Table 7).

**Table 7:** Factor associated with knowledge of UHEPs towards mental illness service provision in Addis Ababa, 2017

Characteristics	Knowledge		COR (95% CI)	AOR (95% CI)
	Adequate n = 200	Inadequate n = 255		
Age group (years)				
20–29	137 (68.5)	201 (78.8)	1.0	1.0
30–44	63 (31.5)	54 (21.1)	0.58 (0.38, 0.89)	0.55 (0.34, 0.90)*
Educational status				
Diploma	133 (66.5)	206 (80.7)	1.0	1.0
Degree	67 (33.5)	49 (19.2)	0.47 (0.31, 0.72)	0.49 (0.32, 0.78)*

*COR* crude odds ratio, *AOR* adjusted odds ratio, *CI* confidence interval

* Statistically significant at P value < 0.05

### Factors associated with attitude towards mental illness

In a bivariate analysis; in-service training, family history and knowledge of mental illness were candidate variables for multivariate logistic regression analysis with P-value < 0.25. Among the variables analyzed for multivariate regression, all the three (in-service training, family history and knowledge) didn’t show significant association with attitude.

### Factors associated with practice of mental health service provision

In a bivariate analysis; age, educational status, work experience, family history of mental illness, supportive supervision, in-service training and job aid were candidate variables for multivariate logistic regression analysis with (P-value < 0.25).

Job aid was statistically significant with practice of UHEP towards provision of mental health service. Those UHEPs who did not have job-aids were four and half times more likely to have good practice comparing to those who had [AOR = (95% CI 4.30 (2.59, 7.15)]. Overall knowledge was also associated with practice; those UHEPs who did not have adequate knowledge were 48% less likely to have good practice compared to those UHEPs who had adequate knowledge with [AOR = 95% CI 0.52 (0.32, 0.85)] (Table 8).

**Table 8:** Factors associated with practice of UHEPs towards mental health service provision in Addis Ababa, 2017

Characteristics	Practice		COR (95% CI)	AOR (95% CI)
	Good n = 342	Poor n = 113		
Do you have job aids on MI				
Yes	200 (58.4)	26 (23.0)	1.0	1.0
No	142 (41.5)	87 (76.9)	4.71 (2.89, 7.67)	4.32 (2.60, 7.18)*
Overall knowledge				
Adequate	163 (47.6)	37 (32.7)	1.0	1.0
Inadequate	179 (52.3)	76 (67.2)	0.53 (0.34, 0.83)	0.52 (0.32, 0.85)*

* Statistically significant at P value < 0.05

## Discussion

This study focused on assessing knowledge, attitude, practice and factors associated with the mental illness service provision among urban health extension professionals in Addis Ababa with a view to suggest an appropriate practice.

According to this study, 44.0% of the study participants had adequate knowledge, when compared to different studies conducted on similar study participants, the result of this study was lower than a study conducted in Addis Ababa, 50% [8], Jimma Zone, 89%, [11] and Chitwan, Nepal, 70% [15]. However, it was higher than the study conducted in Kenya among staffs in general medical facilities which was 34.2% [12]. The possible explanation for this result might be in-service training on mental health is often provided to nurses working in health facilities than urban health extension professionals. This reason is also supported by concepts and recommendation from different literature like WHO, which states that adequate training, skills and competencies are required to effectively assess, diagnose, treat, support and refer people with mental disorder [16]. The key recommendation of mhGap in Ethiopia is to provide training for non-mental health professionals to deliver care for people suffering from mental illness [17]. Again, the Ethiopian mental health strategy, to ensure integration of mental health service into primary health care needs and scaling up the service provision basic and in-service training of health professionals at all levels is very important [18].

Among the study participants involved in this study only 21.5% had a favorable attitude towards mental illness. When compared to other studies, it was lower than the study conducted in Addis Ababa, 44.2% [8], Chitwan, Nepal, 76.5% [11], Nigeria, 43.2% [19], and Kenya, 29.4% [12]. The possible explanation for this result might be the relatively low knowledge of urban health extension professionals on the subject matter, low exposure to mental illness service provision and cultural difference. According to the Ethiopia mental health strategy, such big percentage of unfavorable attitude among UHEPs who are a grass-root level primary health care cadre and who interact with the community on regular basis can negatively affect the already existing cultural believes (the cause of mental illness is supernatural, sprit possession, and the evil eye) of the community and also affect the outcome expected from the integration and scaling up of the mental health service [18]. WHO mental health policy planning and service development also suggest that, stigma can be reduced through primary health care service provider positive attitude towards mental illness. The same literature also indicates the solution to improve primary health care provider`s attitude is training [15].

The overall practice of urban health extension professionals related to service provision for mental illness was 75.2%. This result was aligned with the finding from the study conducted in Kenya on staffs in the general medical facility, referral of suspected patients to mental health care specialist were reported as 89% [12]. Though the overall practice of mental health service provision was good, only 10.8% were diagnosing mental illness at their level. This can be explained from the prospective that low knowledge of UHEPs.

Regarding practicing mental health service provision, about 91% of the study participants were referring the patients with manifestation of mental illness, and larger proportion of UHEPs, 85.3% were providing counseling to the clients and their families. Comparing to the low level of attitude among the study participants, this may be due to lack of basic and refresher training on mental health that inevitably missed and leading to miss management of the patients with mental illness which in turn would adversely affect the outcome of the service provision. The above statement is also supported by WHO mental health policy planning and service development fact sheet which states that low attitude of primary health care providers can affect the human right protection approach [15].

Concerning knowledge, UHEPs whose age were from 30 to 44 years were 45% less knowledgeable when compared to those whose age were 20–29 years. The finding from Addis Ababa Public Hospital showed nurses whose age were 23–27 years two times more knowledgeable than nurses whose age were greater than 37 years [8]. This result was similar to the study conducted in Kenya that among younger nurses (aged 20–30 years), the proportion of those who were “unaware” related to knowledge in psychiatry were higher than older nurses (aged 40 years and above) [12].

Those UHEPs with an educational status of Bachelor degree were 51% less likely to be knowledgeable than those who had diploma. This result was different with the KAP study conducted in Kenya [12], in which the proportion of doctors reported knowledgeable about mental disorders was higher than that of nurses. Similarly, study conducted in Addis Ababa showed that midwifery nurses were four times more knowledgeable than clinical nurses [8].

Urban health extension professionals who did not have a personal history of mental illness were 90% less likely to have adequate knowledge compared to those UHEPs who had personal history. Possible explanation might be those who already had the history might read further about the issues to have better understanding and manage the problem.

Regarding the practice UHEPs, those who did not have job-aids on mental health service provision were four and half times more likely to have good practice compared to those who had a job aid. The possible reason might be those UHEPs who did not have the job aid were using different references that can support their service provision. The overall knowledge was also associated with practice; those UHEPs who did not have adequate knowledge were 48% less likely to have good practice compared to those UHEPs who had adequate knowledge. The possible reason might be those who had knowledge may provide the service easily.

## Limitations

The limitation of this study is the possibility of recall bias that might have been introduced due to some knowledge related questions were difficult to remember because of time. To minimize such bias training on probing techniques has been given to the data collectors and supervisors. The other limitation of this study is lack of published article on the subject to compare our finding with specifically for the factors associated with the disease. Notwithstanding these limitations, we believe that our study has very important findings for strengthening mental health service provision in the study area and areas with similar set up.

## Conclusion

The study found out that more than half urban health extension professionals’ knowledge towards mental illness appear to be inadequate. It is evidence that most of them did not have the opportunity to be exposed to refresher courses related to mental health/illness. In addition to this, a significant proportion of UHEPs in this study had poor attitude towards mental illness. Despite their poor attitude, they happen to had good practice in providing mental health service around referral, follow up and counseling of mentally ill people in the community.

Age, educational status and personal history of MI of urban health extension professionals were associated with knowledge and job-aids and knowledge were associated with mental health service provision. Therefore, provision of basic and refresher trainings to urban health extension professionals is vital to strengthen evidence based medical diagnosis and prevention, also to fully implement the national mental health strategy and maximize the opportunity of the UHEPs at the community level to improve the burden of mental health problem. Moreover, proper clinical supervision and support are also important to improve behavioral change. Besides, further study needs to be conducted to explore how job-aids influence the attitude and practice of UHEPs toward mental illness.

## Abbreviations

FMOH: Federal Ministry of Health
HEP: Health Extension Program
MhGAP: Mental Health Gap Action Program
UHEPs: urban health extension professionals
WHO: World Health Organization
